# Применение клеточных препаратов для лечения критической ишемии нижних конечностей у пациентов с сахарным диабетом: обзор литературы

**DOI:** 10.14341/probl13481

**Published:** 2024-09-15

**Authors:** Г. С. Чуган, А. В. Люндуп, О. Н. Бондаренко, Г. Р. Галстян

**Affiliations:** Национальный медицинский исследовательский центр эндокринологии; Национальный медицинский исследовательский центр эндокринологии; Научно-образовательный ресурсный центр клеточных технологий, Российский университет дружбы народов им. Патриса Лумумбы (РУДН); Национальный медицинский исследовательский центр эндокринологии; Национальный медицинский исследовательский центр эндокринологии

**Keywords:** сахарный диабет, синдром диабетической стопы, критическая ишемия нижних конечностей, ампутация, клеточная терапия, мезенхимальные стромальные клетки, клиническое исследование

## Abstract

За последние десятилетия количество больных сахарным диабетом (СД) во всем мире прогрессивно увеличивается, и многие международные организации рассматривают СД как чрезвычайную ситуацию в здравоохранении XXI века.

Критическая ишемия нижних конечностей (КИНК) является наиболее тяжелой стадией заболеваний артерий нижних конечностей (ЗАНК) при СД и характеризуется высоким риском потери конечности без восстановления кровотока. Традиционные тактики лечения включают открытые и эндоваскулярные методы реваскуляризации. Однако у пациентов, не подлежащих реваскуляризации, и в случаях, когда проведенное хирургическое лечение оказалось недостаточно эффективно, существует мало терапевтических альтернатив, что часто приводит к ампутациям и смерти. На сегодняшний день одним из новейших нехирургических методов лечения является клеточная терапия. В свою очередь мезенхимальные стромальные клетки (МСК) потенциально являются одними из наиболее перспективных для применения у данной категории пациентов.

В представленной статье приведен обзор клинических исследований с использованием клеточной терапии у пациентов с КИНК.

Для анализа публикаций был проведен поиск в электронных базах данных PubMed, SCOPUS, ClinicalTrials и ScienceDirect с целью выявления опубликованных данных клинических испытаний, научных исследований и обзорных статей, посвященных клеточной терапии при критической ишемии нижних конечностей. В результате поиска было получено 489 результатов.

По итогу систематического отбора проведен анализ 22 клинических исследований.

Согласно проанализированным литературным данным, применение клеточных продуктов у данной категории пациентов эффективно и безопасно. Клеточная терапия способствует формированию новых сосудов и усилению коллатерального кровообращения; также отмечается улучшение дистальной перфузии, увеличение дистанции безболевой ходьбы, снижение частоты ампутаций и увеличение коэффициента выживаемости.

Тем не менее необходимо дальнейшее изучение возможностей применения этой категории препаратов.

## Abstract (en)

The number of patients with diabetes mellitus (DM) has been progressively increasing worldwide over the past decades, and many international organizations consider DM as a public health emergency of the 21st century.

Critical limb ischemia (CLI) is the most severe stage of peripheral arterial disease (PAD) in DM and is characterized by a high risk of limb loss without revascularization. Traditional treatment tactics include open and endovascular revascularization surgical techniques. However, in patients not eligible for revascularization and in cases where performed surgical treatment performed has been ineffective, there are almost no therapeutic alternatives, often leading to amputations and death. As of today, one of the newest non-surgical treatment options is cell therapy. Among different cells, mesenchymal stromal cells (MSCs) are potentially one of the most prospective for use in this patient population.

This article provides an overview of clinical trials using cell therapy in patients with CLI.

To analyze publications, electronic databases PubMed, SCOPUS, ClinicalTrials, and ScienceDirect were searched to identify published data from clinical trials, research studies, and review articles on cell therapy for critical lower extremity ischemia. After the search, 489 results were received.

As a result of systematic selection, 22 clinical trials were analyzed.

According to the analyzed literature data, the use of cell products in this category of patients is effective and safe. Cell therapy can stimulate the formation of new vessels and enhances collateral circulation; it is also reported improved distal perfusion, increased pain-free walking distance, decreased amputation rates, and increased survival rates.

Nevertheless, further study of the potential use of this category of drugs is needed.

## ВВЕДЕНИЕ

В 1995 г. Всемирная организация здравоохранения (ВОЗ) объявила СД пандемией неинфекционной природы. С каждым годом количество больных СД во всем мире прогрессивно увеличивается во всех возрастных группах. Общая численность пациентов с СД в Российской Федерации (РФ), состоящих на диспансерном учете, на 01.01.2021 г. составила 4 799 552 (3,23% населения РФ), на 01.01.2022 г. — 4 871 863 (3,35% населения РФ), на 01.01.2023 г. — 4 962 762 человек (3,31% населения РФ) [1].

Синдром диабетической стопы (СДС) считается одним из наиболее серьезных осложнений СД и является следствием патологических изменений периферической нервной системы, дистального сосудистого русла, а также костно-суставного аппарата стопы, что представляет угрозу развития язвенно-некротических процессов и гангрены. Одним из главных факторов риска развития СДС является наличие у пациентов заболеваний артерий нижних конечностей (ЗАНК) [2].

ЗАНК также относятся к числу достаточно частых и наиболее опасных осложнений СД. Частота ЗАНК среди пациентов с СД значительно выше, чем у лиц без нарушений углеводного обмена [2], поскольку перфузия средних и мелких сосудов у пациентов с диабетом уже нарушена. ЗАНК при СД характеризуются малосимптомным либо бессимптомным течением, ранним началом и быстрым прогрессированием атеросклеротических изменений, преобладанием окклюзий артерий над стенозами и высокой постампутационной смертностью [3].

В свою очередь критическая ишемия нижних конечностей (КИНК) представляет собой наиболее тяжелую форму ЗАНК и сопровождается нарушением проходимости магистральных артерий, обычно коррелирующего с прогрессированием атеросклероза. КИНК является независимым фактором риска высокой ампутации у пациентов с СД.

В ряде случаев при КИНК путем хирургического вмешательства не удается достичь значимой реваскуляризации, либо у пациентов изначально имеются противопоказания к оперативному лечению.

На сегодняшний день альтернативным нехирургическим методом лечения у данной категории лиц может стать клеточная терапия [4]. Одним из новейших методов, который активно развивается во всем мире в последние десятилетия, является терапия с применением стромальных клеток.

За последнее десятилетие проведено множество клинических исследований применения клеточных препаратов с различным составом, дозировкой и способами введения.

В данной статье представлен обзор клинических исследований, посвященных применению клеточной терапии для лечения КИНК при СД за последние годы.

## КРИТИЧЕСКАЯ ИШЕМИЯ НИЖНИХ КОНЕЧНОСТЕЙ: ПЕРСПЕКТИВЫ ЛЕЧЕНИЯ ПАЦИЕНТОВ

В настоящее время хирургические методы лечения, такие как эндоваскулярная реваскуляризация, баллонная ангиопластика, установка стентов с лекарственным покрытием, остаются первостепенными в стратегии лечения пациентов с КИНК и СД [4–5]. Важным фактором в послеоперационном периоде для пациентов после реваскуляризации является активное динамическое наблюдение, что позволяет при необходимости провести своевременное повторное вмешательство либо иную коррекцию тактики лечения [6]. Однако, несмотря на то что в большинстве случаев благодаря реваскуляризации удается повысить выживаемость данной категории пациентов, избежать ампутации удается не всегда [7]. К тому же при хирургическом вмешательстве дополнительно возрастает вероятность повреждения сосудов, что в итоге также может привести к увеличению частоты ампутаций [8].

Статистически СД является основной причиной потери нижних конечностей во всем мире. Ежегодно более миллиона пациентов с СД подвергаются ампутации нижних конечностей [9]. На сегодняшний день высокие ампутации нижних конечностей в РФ приходится выполнять с частотой до 25% у пациентов с критической ишемией на фоне облитерирующего атеросклероза магистральных артерий нижних конечностей и до 50% — при распространенном гнойно-некротическом поражении тканей у больных с СДС [10].

В связи с этим возникает потребность в нехирургических методах лечения, одним из которых является клеточная терапия. Во всем мире ведется поиск альтернатив для пациентов без возможности проведения реваскуляризации. Во многих публикациях авторы приходят к выводу, что терапевтическое лечение, в том числе применение генных и клеточных препаратов, снижает частоту ампутаций у таких пациентов за счет улучшения артериальной перфузии и заживления ран [11]. В связи с острой необходимостью новых методов лечения проводится большое количество исследований по безопасности и эффективности клеточной терапии. В недавнем обзоре рандомизированных контролируемых испытаний (РКИ) и метаанализов (МА) на основании изученного материала Shi H. et al. пришли к выводу, что применение стволовых клеток у пациентов с СДС эффективно и безопасно [12].

Среди различных препаратов клеточной терапии мезенхимальные стромальные клетки (МСК) обладают наибольшим потенциалом в лечении различных заболеваний, в том числе диабетических язв и КИНК при СД, благодаря их роли в регенерации тканей путем стимуляции ангиогенеза и иммуномодуляции [13–16]. В 2017 г. Максимовой Н.В. и соавт. в рамках клинического исследования была доказана эффективность аутологичных МСК в заживлении диабетических язв [17]. Также в рамках обзора клинических исследований применения МСК для лечения диабетических язв в 2022 г. Красильникова О.А. и соавт. пришли к выводу, что местное применение МСК способствует только заживлению язвенных дефектов, в то время как внутримышечные и внутриартериальные инъекции МСК или мононуклеарных клеток могут разорвать патофизиологическую цепочку, ведущую от недостаточного кровоснабжения к развитию трофических изменений [18].

По данным большого количества проведенных исследований, установлена безопасность и целесообразность терапии аутологичными и аллогенными МСК [19], однако многие авторы приходят к выводу о важности проведения новых исследований для оптимизации процедуры лечения, а также для того, чтобы иметь возможность предложить новое поколение стволовых клеток, которые можно было бы регулярно использовать в экономически эффективной и безопасной терапии, направленной на лечение КИНК при СД.

На сегодняшний день уже существуют зарегистрированные препараты мезенхимальных стромальных клеток в Индии и Германии, которые активно используются в лечении пациентов с различными хроническими заболеваниями, в том числе с СД и КИНК [20–21]. Также в настоящее время в мире проводится ряд регистрационных и пилотных академических исследований новых клеточных препаратов.

Целью данной статьи является анализ проведенных клинических исследований применения клеточной терапии для лечения КИНК при СД для оценки данного метода лечения в долгосрочной перспективе.

## МАТЕРИАЛЫ И МЕТОДЫ

Для анализа публикаций был проведен поиск в электронных базах данных PubMed, SCOPUS, ClinicalTrials и ScienceDirect с целью выявления опубликованных данных клинических испытаний, научных исследований и обзорных статей, посвященных применению стромальных и мононуклеарных клеток для лечения КИНК. В результате поиска было получено 489 результатов. Из них было исключено 65 незавершенных исследований. В результате прочтения заголовков и аннотаций из 424 статей было отобрано 51 исследование. Из них после полного прочтения по различным причинам (неподходящий диагноз, неподходящая модель проведения исследования, неподходящий состав клеточного препарата и др.) было исключено 29 исследований. Оставшиеся 22 клинических исследования были включены в данный обзор.

Механизм отбора публикаций для включения в обзор представлен на рис. 1.

**Figure:**
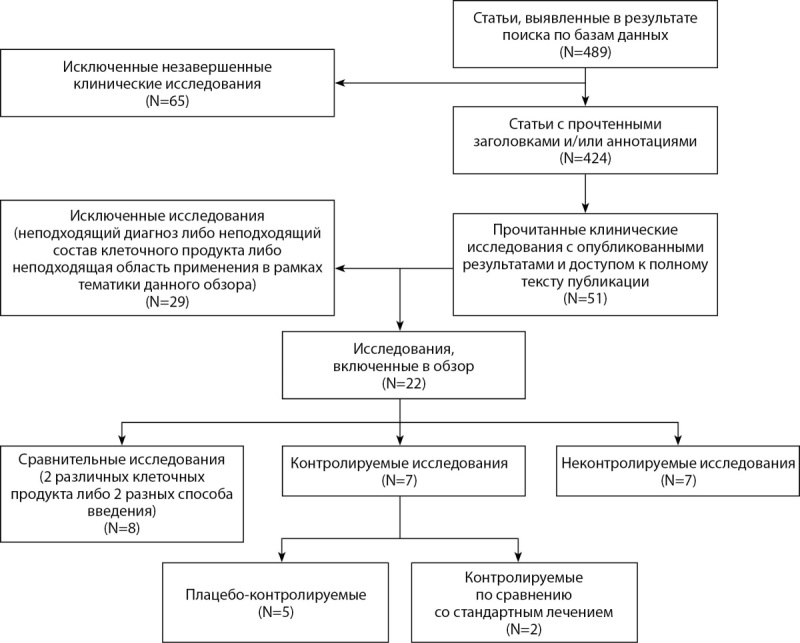
Рисунок 1. Блок-схема отбора статей для дальнейшего анализа.

Данные о проведенных клинических исследованиях с использованием клеточной терапии представлены в табл. 1 (Приложение 1) [21-40].

## ОБСУЖДЕНИЕ

Во всех обозреваемых исследованиях применение клеточных препаратов было эффективно, что подтверждается улучшением таких инструментальных показателей, как лодыжечно-плечевой индекс (ЛПИ), транскутанная оксиметрия (TcO2), а также увеличением выживаемости пациентов и уменьшением количества ампутаций и летальных исходов на фоне групп контроля, получавших плацебо либо терапию в соответствии со стандартами лечения.

Увеличение ЛПИ у пациентов отмечено в 8 исследованиях, в 1 исследовании у части пациентов наблюдалось снижение ЛПИ, в 2 исследованиях наблюдалось увеличение ЛПИ в исследуемой группе на фоне отсутствия изменений в группе плацебо за весь период наблюдений. Общее улучшение показателей TcO2 у пациентов отмечено в 10 исследованиях.

В половине работ (11 из 22) в группах исследования отмечена значительная роль клеточных препаратов в ранозаживлении: в большинстве исследований количество пациентов с полным или частичным заживлением язвенных дефектов составило от 60 до 100% [21–23][25][29][31][37–39], в то время как в контрольных группах за тот же период наблюдений данные показатели варьируют от 0 до 13% [30–31][36].

В обозреваемых исследованиях также отмечено снижение выраженности болевого синдрома в пораженных конечностях у 70–100% пациентов в группах исследования [25][31–32][37–38][40].

При сравнении общей выживаемости и количества ампутаций у пациентов, получавших клеточные препараты, частота ампутаций и летальных исходов в среднем оказалась на 10% ниже по сравнению с контрольными группами [30][36]. Количество ампутаций и летальных исходов в группах исследования составило от 0 до 40% в течение всего периода наблюдения (в разных исследованиях от 6 месяцев до 10 лет) [27][29–30][32–34][36–37][39].

Согласно проанализированным данным, а также проведенным ранее исследованиям [41], эффективность клеточной терапии может быть выявлена как за короткий промежуток времени после применения клеточного продукта, так и в долгосрочной перспективе. Однако в исследованиях с длительным периодом наблюдения было установлено, что однократного введения клеточного препарата может быть недостаточно для поддержания достигнутых улучшений кровоснабжения пораженной конечности в течение длительного времени [32][34][38–39].

В большинстве исследований (17 из 22) в качестве оценки результата лечения была выбрана выживаемость пациентов за период наблюдений, наличие ампутаций и динамика изменения язвенных дефектов. Эти исходы, на наш взгляд, являются одними из наиболее важных для оценки эффективности клеточных препаратов.

В 14 из 22 исследований в качестве критерия включения пациентов была установлена невозможность проведения реваскуляризации, что в очередной раз подчеркивает важность поиска нехирургических методов лечения данной категории пациентов с КИНК и, на наш взгляд, является одним из основных показаний для применения клеточной терапии. В качестве показания к применению клеточных препаратов также следует рассматривать наличие длительно незаживающих язвенных дефектов [12][17].

В некоторых исследованиях были использованы лабораторные маркеры в качестве конечных точек из-за легкости в получении результатов за довольно короткий промежуток наблюдений [42], однако данные исходы не всегда коррелируют с общим состоянием и исходами лечения пациентов. Поэтому, на наш взгляд, для более точной оценки безопасности и эффективности клеточных препаратов необходимо расширить спектр оцениваемых показателей.

В обозреваемых сравнительных исследованиях не было получено статистически значимых различий в исходах у пациентов в зависимости от способа введения препарата [28–29]. В связи с этим мы полагаем, что внутримышечный способ введения стромальных клеток является предпочтительным по сравнению с внутриартериальным введением, ввиду большей безопасности и меньшего риска развития нежелательных реакций.

Всего в трех исследованиях [24][25][28] большое внимание уделялось оценке развития у пациентов коллатерального кровотока и неоангиогенеза. В то же время инструментальные методы исследования, на наш взгляд, являются важным аспектом в динамическом наблюдении данной категории пациентов. Одним из таких методов может стать оценка транскутанной оксиметрии в динамике, также возможно применение дополнительных методов визуализации [43-45]. Для дополнительной оценки объемной скорости кровотока можно рассмотреть возможность проведения КТ-ангиографии нижних конечностей [24] либо аналогичные методы визуализации коллатерального кровотока у исследуемых групп пациентов.

Имеющиеся результаты доказательной медицины свидетельствуют о безопасности, минимальном количестве осложнений и нежелательных явлений и эффективности клеточной терапии [46–47], что открывает новые возможности для дальнейшего исследования данного метода лечения.

## ЗАКЛЮЧЕНИЕ

Клеточные технологии могут стать перспективным направлением в лечении пациентов с СД и КИНК в случаях, когда традиционные методы восстановления кровотока имеют ограничения. Проанализированные данные позволяют утверждать о безопасности и эффективности применения клеточной терапии у пациентов с КИНК при СД. На сегодняшний день в мире зарегистрировано 2 препарата для лечения ран и КИНК у больных СД, а также проводится ряд регистрационных исследований в этом направлении. Однако необходимы дальнейшие исследования в более крупных когортах для подтверждения этих данных и проверки этого метода лечения у других пациентов. Тем не менее описанные результаты являются примером успешного ведения пациентов с КИНК в долгосрочной перспективе и имеют большое клиническое значение, поскольку спасение конечностей, снижение смертности и улучшение качества жизни являются основной целью лечения нейроишемической формы СДС.

## ДОПОЛНИТЕЛЬНАЯ ИНФОРМАЦИЯ

Источники финансирования. Статья подготовлена на основании результатов, полученных в ходе реализации Соглашения о предоставлении гранта в форме субсидий из федерального бюджета на осуществление государственной поддержки создания и развития научных центров мирового уровня, выполняющих исследования и разработки по приоритетам научно-технологического развития от 20 апреля 2022 г. № 075-15-2022-310.

Конфликт интересов. Авторы декларируют отсутствие явных и потенциальных конфликтов интересов, связанных с публикацией статьи.

Участие авторов. Все авторы одобрили финальную версию статьи перед публикацией, выразили согласие нести ответственность за все аспекты работы, подразумевающую надлежащее изучение и решение вопросов, связанных с точностью или добросовестностью любой части работы.

## ПРИЛОЖЕНИЕ №1.

**Table:** Таблица 1. Обзор клинических исследований с применением клеточных препаратов при КИНК и СД Аббревиатуры: КИНК — критическая ишемия нижних конечностей; СД — сахарный диабет; Ds — диагноз; N — количество пациентов, включенных в исследование; в/м — внутримышечно; в/а — внутриартериально; в/в — внутривенно; MSC — мезенхимальные стромальные клетки; ЛПИ — лодыжечно-плечевой индекс; TcPO2 — транскутанное напряжение кислорода; сBMA — концентрированный аспират костного мозга; BMMSCs — мезенхимальные стромальные клетки костного мозга; BMMNCs — мононуклеарные клетки, полученные из костного мозга; P-MSCs — мезенхимальные стромальные клетки, полученные из плацентарной ткани; BMCs — клетки косного мозга; WJ-MSCs — мезенхимальные стромальные клетки, полученные из желе Вартона; BM-CTPs — клеточные продукты, полученные из костного мозга; PB-CTPs — клеточные продукты, полученные из периферической крови; PCCs — очищенные CD34+ клетки; PBMNCs — мононуклеарные клетки, полученные из периферической крови; EPC-CFU — колониеобразующие единицы эндотелиальных прогениторных клеток.

№	Пациенты (Ds, N)	Критерии включения, сроки проведения исследования	Тип клеток, методы введения, дозировка	Результаты	NCT/ DOI
1 [21]	КИНК N=24	1) Установленный диагноз КИНК. 2) ЛПИ <0,60. 3) Наличие как минимум одного язвенного дефекта. 4) Компенсация СД. Сроки проведения: 2018–2023 гг.	Stempeucel® (MSC) В/м введение в икроножную мышцу и вокруг язвенного дефекта Дозировка: 2 млн клеток/кг массы тела	За 1 год наблюдений: •23 язвы (82,1%) зажили полностью, •4 язвы (14,3%) зажили частично. (p<0,0001). Среднее систолическое давление в лодыжке увеличилось с 61 мм рт. ст. на исходном уровне до 81 мм рт. ст. через 1 мес, 89 мм рт. ст. через 3 мес, 94 мм рт. ст. через 6 мес и 95 мм рт. ст. через 12 мес. (p<0,0001). Среднее значение ЛПИ увеличилось с 0,47 мм рт. ст. на исходном уровне до 0,61 мм рт. ст. через 1 мес, 0,67 мм рт. ст. через 3 мес, 0,70 мм рт. ст. через 6 мес и 0,73 мм рт. ст. через 12 мес. (p<0,0001)	DOI: 10.1186/s13287-023-03292-w
2	КИНК N=153	1) Установленный диагноз КИНК. 2) Невозможность проведения реваскуляризации. 3) ЛПИ≤0,6, или TcPO2 ≤50 мм рт. ст. Сроки проведения: 2010–2020 гг.	сBMA В/м введение в пораженную конечность Точная дозировка клеток не указана	За 1 год наблюдений: •исследуемая группа — 20,2% высоких ампутаций или летальных исходов (24/119); •группа контроля (плацебо) — 30,6% (11/36). За 5 лет наблюдений: •исследуемая группа — 30,3% высоких ампутаций или летальных исходов (36/119); •группа контроля (плацебо) — 44,4% (16/36)	01049919
3	СД 2 типа N=16	1) Наличие СД2 HbA1c менее 7,0%). 2) Наличие эндотелиальной дисфункции. Сроки проведения: 2017–2020 гг.	MSC В/в введение Дозировка: 20 или 100 млн клеток	За 1 год наблюдений: •100% выживаемость без ампутаций. Уровень колониеобразующих единиц эндотелиальных прогениторных клеток (EPC-CFU) возрос с 2.06-3.13 до 6.89-8.83	02886884
4 [22]	КИНК СД2 N=41	1) Установленный диагноз КИНК и СД2. 2) ЛПИ от 0,30 до 0,60. 3) Наличие как минимум одного язвенного дефекта. Сроки проведения: 2009–2011 гг.	BMMSCs BMMNCs В/м введение Дозировка: 0,5–2,0 мл клеточного концентрата	За 6 мес наблюдений: •в группе BMMSC — увеличение среднего ЛПИ с 0,55 до 0,72; увеличение TcO2 с 45 до 66 мм рт. ст.; 100% заживлений язвенных дефектов за 8 недель (p<0,05); •в группе BMMNC — увеличение среднего ЛПИ с 0,55 до 0,63; увеличение TcO2 с 45 до 60 мм рт. ст.; 100% заживлений язвенных дефектов за 12 недель (p<0,05); •в группе плацебо — отсутствие увеличения среднего ЛПИ (0,55); отсутствие увеличения TcO2; 80% заживлений язвенных дефектов за 24 недели (p<0,05)	00955669 DOI: 10.1016/j.diabres.2010.12.010
5 [23]	КИНК N=20	1) Установленный диагноз КИНК. 2) Невозможность проведения реваскуляризации. 3) ЛПИ ≤0,6, или TcPO2 ≤60 мм рт. ст. 4) Компенсация СД. Сроки проведения: 2009–2013 гг.	BM-MSCs В/м введение Дозировка: 200 млн клеток.	За 6 мес наблюдений в исследуемой группе отмечено значительное увеличение уровня ЛПИ (с 0,55 до 0,78) по сравнению с группой плацебо (0,60–0,60). (p=0,0018). За 2 года наблюдений отмечено 100% уменьшения в размерах либо полного заживления язвенных дефектов в обеих группах	00883870 DOI: 10.1186/1479-5876-11-143
6 [24]	КИНК СД N=20	1) Установленный диагноз КИНК и СД. 2) Невозможность проведения реваскуляризации. Сроки проведения: 2007–2011 гг.	BMMNC В/а введение Дозировка: 100–400 млн клеток	За 3 мес наблюдений: •по данным ангиографии у всех пациентов отмечена значительная неоваскуляризация с развитием коллатерального кровообращения в пораженной области. Также отмечено увеличение среднего ЛПИ с 0,46±0,19 до 0,70±0,23 (p<0,01)	00872326 DOI: 10.3727/096368910X0177
7 [25]	КИНК N=11	1) Установленный диагноз КИНК. 2) Невозможность проведения реваскуляризации. Сроки проведения: 2021–2023 гг.	P-MSCs В/м в два этапа с интервалом в 8 недель Дозировка: 20 или 60 млн клеток	За 6 мес наблюдений: •полный цикл наблюдений завершили 8 пациентов; •100% пациентов (8/8) отмечали уменьшение болевого синдрома; •у 75% пациентов (6/8) увеличена дистанция безболевой ходьбы; •у 62,5% пациентов (5/8) отмечено увеличение ЛПИ; •у 25% (2/8) отмечено снижение ЛПИ; •у 87,5% пациентов (7/8) отмечено заживление трофических язв и некротических дефектов. Также описано увеличение коллатерального кровотока за счет неоангиогенеза у всех пациентов (p<0,0001)	DOI: 10.1186/s13287-023-03390-9
8 [26]	КИНК N=9	1) Установленный диагноз КИНК. 2) Невозможность проведения реваскуляризации. 3) Предполагаемая продолжительность жизни >6 мес. Сроки проведения: 2013–2020 гг.	BM-MSCs 20 в/м инъекций по 0,5 мл Дозировка: 20, 40 или 80 млн клеток	За 1 год наблюдений: •у 4/9 пациентов удалось достичь полного заживления язвенного дефекта (у 3 из них — без дальнейших ампутаций); •у 3/9 пациентов не удалось адекватно оценить результаты лечения в связи с выявленными аномальными кариотипами в мезенхимальных стромальных клетках	DOI: 10.1016/j.jcyt.2020.02.007
9 [27]	КИНК N=60	1) Установленный диагноз КИНК. 2) Невозможность проведения реваскуляризации. Сроки проведения: 2008–2012 гг.	BMА В/а введение в два этапа с интервалом 45 дней Дозировка: 100 мл аспирата костного мозга	За 1 год наблюдений: •лазерная допплеровская базальная оксигенация: Т0 — 65,095±53,3 против Т12 — 205,03±114,39; •TcPO2: Т0 — 26,96±15,83 против Т12 — 36,13±22,47 мм рт.ст. (p=0,008) За рассматриваемый период произошло 8 высоких ампутаций (16,6%) и 4 смерти (8,3%)	DOI: 10.5966/sctm.2012-0021
10 [28]	КИНК N=41	1) Установленный диагноз КИНК. 2) Невозможность проведения реваскуляризации. 3) ЛПИ ≤0,4, или TcPO2 ≤30 мм рт. ст. Сроки проведения: 2009–2012 гг.	BMCs 21 пациент — в/м введение; 20 пациентов — в/а введение Дозировка: 40 мл клеточного концентрата	За 6 мес наблюдений: •в обеих группах было отмечено значимое увеличение уровня TcPO2 (с 15±10 мм рт. ст. до 29±13 мм рт. ст.) (p<0,001). По данным цифровой субтракционной ангиографии, не было выявлено заметного развития новых коллатеральных сосудов через 6 мес по сравнению с исходными ангиограммами	DOI: 10.3727/096368912X636948
11 [29]	КИНК N=62	1) Установленный диагноз КИНК. 2) Невозможность проведения реваскуляризации. Сроки проведения: 2012–2016 гг.	BMCs 32 пациента — в/м введение; 30 пациентов — в/а введение Дозировка: 40 мл клеточного концентрата	За 1 год наблюдений: •7/62 (11%) летальных исходов; •39/62 (63%) сохранения конечности без ампутации; •33/55 (60%) случаев заживления язвенных дефектов. Также отмечено увеличение уровня TcPO2 с исходных 16±10 до 27±14 (p<0,001)	DOI: 10.1186/s13287-016-0379-z
12 [30]	КИНК N=59	1) Установленный диагноз КИНК. Сроки проведения: 2007–2011 гг.	BMCs В/м введение Дозировка: 136±41 млн клеток	За 1 год наблюдений: •исследуемая группа (32 пациента, завершивших цикл наблюдений) — 6 высоких ампутаций (19%); 1 летальный исход (3%); 6 случаев ухудшения/появления новых язвенных дефектов (19%); 31% полного заживления язвенных дефектов (p=0,038); •группа контроля (14 пациентов, завершивших полный цикл наблюдений) — 6 высоких ампутаций (43%); 1 летальный исход (7%); 6 случаев ухудшения/появления новых язвенных дефектов (43%); 13% полного заживления язвенных дефектов (p=0,038)	DOI: 10.1016/j.jvs.2011.04.006
13 [31]	КИНК СД N=24	1) Установленный диагноз КИНК и СД. Сроки проведения: 2019–2022 гг.	auto-BM-MNC Allo-WJ-MSCs В/м введение Дозировка: auto-BM-MNC — 15 инъекций по 7,197±2,984 млн клеток Allo-WJ-MSCs — 15 инъекций по 1,333 млн клеток	За 1 год наблюдений: •заживление язвенных дефектов — различия между auto-BM-MNC и Allo-WJ-MSCs по сравнению с группой плацебо (90,74±20,70% против 92,68±16,76% против 2±4,47% соответственно); •TcPO2 — различия между auto-BM-MNC и Allo-WJ-MSCs по сравнению с группой плацебо (47,50±15,02 мм рт. ст. против 65±13,21 мм рт. ст. против 1,88±4,37 мм рт. ст. соответственно). Дистанция безболевой ходьбы — различия между группами auto-BM-MNC и Allo-WJ-MSCs по сравнению с группой плацебо (850±1061 м против 306±225 м против 3,75±7,44 м соответственно) (p<0,05)	05631444 DOI: 10.1186/s13287-023-03427-z
14 [32]	КИНК СД2 N=41	1) Установленный диагноз КИНК и СД2. 2) ЛПИ от 0,30 до 0,60. 3) Наличие как минимум одного язвенного дефекта. Сроки проведения: 2009–2019 гг.	BMMSCs BMMNCs В/м введение Дозировка: 0,5–2,0 мл клеточного концентрата	Спустя 6 месяцев наблюдений: •количество ампутаций: 7 (23,3%) в группе BMMSC, 8 (26,7%) — в группе BMMNC и 15 (50,0%) — в контрольной группе (p<0,05); •частота рецидивов язвы в группе BMMSC была значительно ниже, чем в контрольной группе в течение 3–6 мес после заживления язвы (спустя 3 мес: 0/11 в группе BMMSC против 6/16 в контрольной группе; спустя 6 мес: 3/11 в группе BMMSC против 11/16 в контрольной группе) (p<0,05) Однако многие оцениваемые показатели (боли в покое, временя безболевой ходьбы, ЛПИ, TcO2 и ангиографические показатели), также демонстрировавшие положительную динамику в течение первых 6–9 мес наблюдений, по итогам 3 лет наблюдений вернулись на уровень исходных значений (p<0,05)	DOI: 10.1177/0963689719835177
15 [33]	КИНК N=40	1) Установленный диагноз КИНК. 2) Невозможность проведения реваскуляризации. Сроки проведения: 2007–2017 гг.	BM-CTPs PB-CTPs В/м введение Дозировка: 30 в/м инъекций по 1 мл клеточного концентрата в каждой	За 6 мес наблюдений: •группа BM-CTP — 40% высоких ампутаций или летальных исходов (8/20); Группа PB-CTPs — 30% (6/20) (p<0.05); •в группе PB-CTP удалось добиться повышения TcPO2 в 2 раза по сравнению с группой BM-CTP, где не было отмечено статистически значимых изменений (p<0,05)	00533104 DOI: 10.1016/j.jcyt.2016.10.013
16 [34]	КИНК N=50	1) Установленный диагноз КИНК. Сроки проведения: 2007–2019 гг.	BM-MNCs В/м введение Дозировка: 0,5 мл клеточного концентрата	За 10 лет наблюдений: •общая выживаемость без ампутаций составила 73,0% через 5 лет и 70,4% через 10 лет у пациентов из группы BM-MNC	DOI: 10.1038/s41598-019-44176-5
17 [35]	КИНК N=148	1) Установленный диагноз КИНК. Сроки проведения: 2009–2021 гг.	PBMNCs PCCs В/м введение Дозировка: 0,5 мл клеточного концентрата	За 1 год наблюдений: •исследуемая группа — 12,2% рецидивов КИНК или выявлений новых поражений конечностей (18/148) (p<0,04)	DOI: 10.1093/stcltm/szac017
18 [36]	КИНК N=58	1) Установленный диагноз КИНК. 2) Невозможность проведения реваскуляризации. Сроки проведения: 2008–2011 гг.	BM-MNCs В/м введение Дозировка: 50–120 мл клеточного концентрата	За 6 мес наблюдений: •исследуемая группа — 5/19 (26%) язв зажили полностью или частично; •группа контроля (плацебо) — 0/17 (0%) язв зажили полностью или частично (p<0,05); •исследуемая группа — 3/29 (10%) высоких ампутаций; •группа контроля (плацебо) — 5/29 (17%) высоких ампутаций (p<0,05)	DOI: 10.6002/ect.2012.0129
19 [37]	КИНК СД N=50	1) Установленный диагноз КИНК и СД. 2) Невозможность проведения реваскуляризации. 3) TcPO2<0,60 мм рт. ст. Сроки проведения: 2018-2021 гг.	PB-MNCs В/м введение Дозировка: концентрат из 120 мл периферической крови	За 1 год наблюдений: •у 60% пациентов (30/50) отмечено уменьшение или полное заживление язвенного дефекта; •16 пациентов (32%) погибло, 8 пациентов (16%) перенесло высокую ампутацию; •16 (47% от выживших) полностью вылечились без высокой ампутации, 26 (88,5% от выживших) отмечали уменьшение или исчезновение болей и улучшение качества жизни (p<,0001)	DOI: 10.1186/s12933-022-01629-y
20 [38]	КИНК N=160	1) Установленный диагноз КИНК. 2) Невозможность проведения реваскуляризации. 3) Наличие язвенного дефекта, не заживающего после как минимум 1 месяца активного лечения. Сроки проведения: 2009–2019 гг.	PBMNCs PCCs В/м введение Дозировка: 0,5 мл клеточного концентрата	За 5 лет наблюдений: •у 100% пациентов (47/47, завершивших 5-летний цикл), отмечено полное заживление язвенных дефектов; •у 98% пациентов (47/48, завершивших 5-летний цикл) отмечено улучшение состояния конечности (по классификации Резерфорда 0–3). Также в обеих исследуемых группах отмечено прогрессивное улучшение ЛПИ и TcPO2 (p<0,01)	DOI: 10.1186/s13287-020-01981-4
21 [39]	КИНК N=52	1) Установленный диагноз КИНК. 2) Невозможность проведения реваскуляризации. Сроки проведения: 2014–2019 гг.	PBMNCs PCCs В/м введение Дозировка: 0,5 мл клеточного концентрата	За 3 года наблюдений: •группа PBMNC — 4/25 (16%) ампутаций, 0% летальных исходов, 21/24 (87,5%) случай заживления язвенных дефектов. (p<0,05); •группа PCC — 8/25 (32%) ампутаций, 2/25 (8%) летальных исхода, 22/24 (91,7%) случая заживления язвенных дефектов(p<0,05). Спустя 2 года от начала исследования в группе PBMNC отмечено значимое увеличение ЛПИ на фоне группы PCC, однако спустя 3 года от начала исследований показатели ЛПИ в обеих группах оказались сопоставимы (p<0,05). В обеих исследуемых группах за первый год наблюдений отмечено увеличение TcPO2, однако спустя 3 года наблюдений показатели мало отличались от исходных значений (p<0,05)	DOI: 10.1002/sctm.20-0033
22 [40]	КИНК N=50	1) Установленный диагноз КИНК. 2) Невозможность проведения реваскуляризации. 3) ЛПИ ≤0,4, TcPO2 ≤30 мм рт. ст. 4) Наличие как минимум одного язвенного дефекта или гангрены. Сроки проведения: 2016–2017 гг.	BM-MNC 25 пациентов — в/м введение; 25 пациентов — в/а введение Дозировка: 40 мл концентрата костного мозга	За 6 мес наблюдений: •в обеих группах было отмечено значимое увеличение уровня TcPO2 (с 16±10 мм рт. ст. до 29±14 мм рт. ст.), уменьшение симптомов по шкале боли 0–10 (с 4,4±2,4 до 1,6±1,6) (p<0,05)	DOI: 10.1186/s13287-017-0622-2
